# PS-17 - THE EMERGENCE OF BRUCELLA CANIS IN ENGLAND: A NEW CHALLENGE FOR HUMAN BRUCELLOSIS SURVEILLANCE

**DOI:** 10.1093/pubmed/fdag046.064

**Published:** 2026-07-09

**Authors:** Dr Charlotte Robin, Katie Harman, Dr Dimple Chudasama, Dr Alessandro Gerada, Jessica Jervis, Dr Stephen Woolley, Dr Rachel Pudney

**Affiliations:** UK Health Security Agency; UK Health Security Agency; UK Health Security Agency; Brucella Reference Unit; Brucella Reference Unit; Brucella Reference Unit; UK Health Security Agency

## Abstract

**Background:**

Human brucellosis caused by *Brucella canis* is rare and is primarily transmitted through direct contact with reproductive fluids from an infected dog. Current diagnostics for *Brucella* species include culture, smooth-strain species serology and a pan-*Brucella* PCR assay. No accredited serological assays exist for *Brucella canis* in humans.

**Objectives:**

To describe trends and challenges of brucellosis surveillance in England.

**Methods:**

National surveillance data between 2017 and 2025 were obtained through the UKHSA laboratory reporting system (SGSS).

**Results:**

On average, 15 cases of brucellosis are reported each year, primarily travel-related *Brucella melitensis*. Since 2022, numbers have increased, due to; changes in travel; emergence of *B. canis*; changes in notification practices. Following an increase in PCR-positive results linked to *B. canis*–positive dogs, laboratory results from 2022 onwards are interpreted alongside clinical and epidemiological information and allocated a case definition.

**Challenges:**

Key challenges include the inability of the pan-*Brucella* PCR to determine species and the absence of accredited serological *B. canis* diagnostics, both of which limit interpretation of laboratory results. Only *Brucella* species PCR and non-canis serology results are captured in SGSS, meaning comprehensive surveillance for all *Brucella* species cannot be achieved using SGSS alone. SGSS data often lacks sufficient clinical context to interpret PCR results, and laboratory request forms frequently provide minimal exposure or clinical information.

**Conclusion:**

Brucellosis remains rare in England, but the emergence of *B. canis* and limitations of current diagnostics and surveillance highlight gaps in national monitoring. Current surveillance means we are unable to systemically monitor *B. canis* as an emerging infection. Improving diagnostic capability, including accredited serological assays and PCR for *B. canis*, expanding the range of data captured through SGSS, and strengthening integration between clinical, laboratory, and public health teams will be essential to ensure timely identification, accurate case classification, and effective public health follow-up.

